# Comparing the associations of central venous pressure and pulmonary artery pulsatility index with postoperative renal injury

**DOI:** 10.3389/fcvm.2022.967596

**Published:** 2022-10-12

**Authors:** Johnny Wei, Abigail Houchin, Niaman Nazir, Vincent Leonardo, Brigid C. Flynn

**Affiliations:** ^1^Department of Anesthesiology, University of Kansas Medical Center, Kansas City, KS, United States; ^2^Department of Population Health, University of Kansas Medical Center, Kansas City, KS, United States

**Keywords:** AKI, CVP, PAPi, cardiac surgery, critical care

## Abstract

**Objective:**

Cardiac surgery-associated acute kidney injury (CS-AKI) is associated with significant morbidity and mortality. We investigated the association of postoperative central venous pressure (CVP) and pulmonary artery pulsatility index (PAPi) with the development of CS-AKI.

**Methods:**

This was a single-center, retrospective cohort study of patients undergoing cardiac surgery. CVP and PAPi were acquired hourly postoperatively and averaged for up to 48 h. PAPi was calculated as [(Pulmonary Artery Systolic Pressure–Pulmonary Artery Diastolic Pressure) / CVP]. The primary aim was CS-AKI. Secondary aims were need for renal replacement therapy (RRT), hospital and 30-day mortality, total ventilator and intensive care unit hours, and hospital length of stay. Logistic regression was used to calculate odds of development of renal injury and need for RRT.

**Results:**

One thousand two hundred eighty-eight patients were included. The average postoperative CVP was 10.3 mmHg and average postoperative PAPi was 2.01. Patients who developed CS-AKI (*n* = 384) had lower PAPi (1.79 vs. 2.11, *p* < 0.01) and higher CVP (11.5 vs. 9.7 mmHg, *p* < 0.01) than those who did not. Lower PAPi and higher CVP were also associated with each secondary aim. A standardized unit decrease in PAPi was associated with increased odds of CS-AKI (OR 1.39, *p* < 0.01) while each unit increase in CVP was associated with both increased odds of CS-AKI (OR 1.56, *p* < 0.01) and postoperative RRT (OR 1.49, *p* = 0.02).

**Conclusions:**

Both lower PAPi and higher CVP values postoperatively were associated with the development of CS-AKI but only higher CVP was associated with postoperative RRT use. When differences in values are standardized, CVP may be more associated with development of CS-AKI when compared to PAPi.

## Introduction

Acute kidney injury (AKI) occurs in ~25% of patients following cardiac surgery and is associated with significant morbidity and mortality (1–3). The etiology of cardiac surgery-associated AKI (CS-AKI) is multifactorial and results from a combination of underlying disease burden and perioperative insults (1–4). An increasingly recognized contributor to CS-AKI is right ventricular (RV) dysfunction which decreases renal perfusion due to elevated central venous pressure (CVP) (5–7). Increased CVP has been associated with renal dysfunction even independent of cardiac output, and remains an important focus of perioperative care in cardiac surgical patients (8–10). Although the utility of CVP as a marker of volume status and venous congestion has been called into question (11), recent evidence has supported the association of higher CVP and CS-AKI secondary to increased inflammation and sympathetic activation (10, 12, 13).

The pulmonary artery pulsatility index (PAPi) is a novel hemodynamic measurement of RV function defined as the pulmonary artery pulse pressure divided by CVP (14–16). Though originally used to assess RV failure following acute myocardial infarction (15), decreased PAPi has been proposed to predict postoperative RV dysfunction after major cardiac surgery such as heart transplantation and ventricular assist device implantation (7, 17–20). There is also evidence that the development of RV dysfunction and resultant venous congestion contributes to renal dysfunction following cardiac surgery and has made PAPi a new area of recent investigation (5, 6, 21).

Due to lack of robust literature specific to postoperative cardiac surgical patients, we sought to compare the associations of postoperative PAPi and CVP to CS-AKI in this patient population. Furthermore, we also sought to compare which of the two hemoynamic parameters has a stronger association with CS-AKI.

## Materials and methods

This was a single-center retrospective cohort study of adult patients who underwent cardiac surgery between January 1^st^, 2017 and December 31^st^, 2019 at a tertiary-care academic medical center. This study was approved for a quality improvement designation and considered exempt from review by the University of Kansas institutional review board, and the requirement for written informed consent was consequently waived.

**Figure 1:**
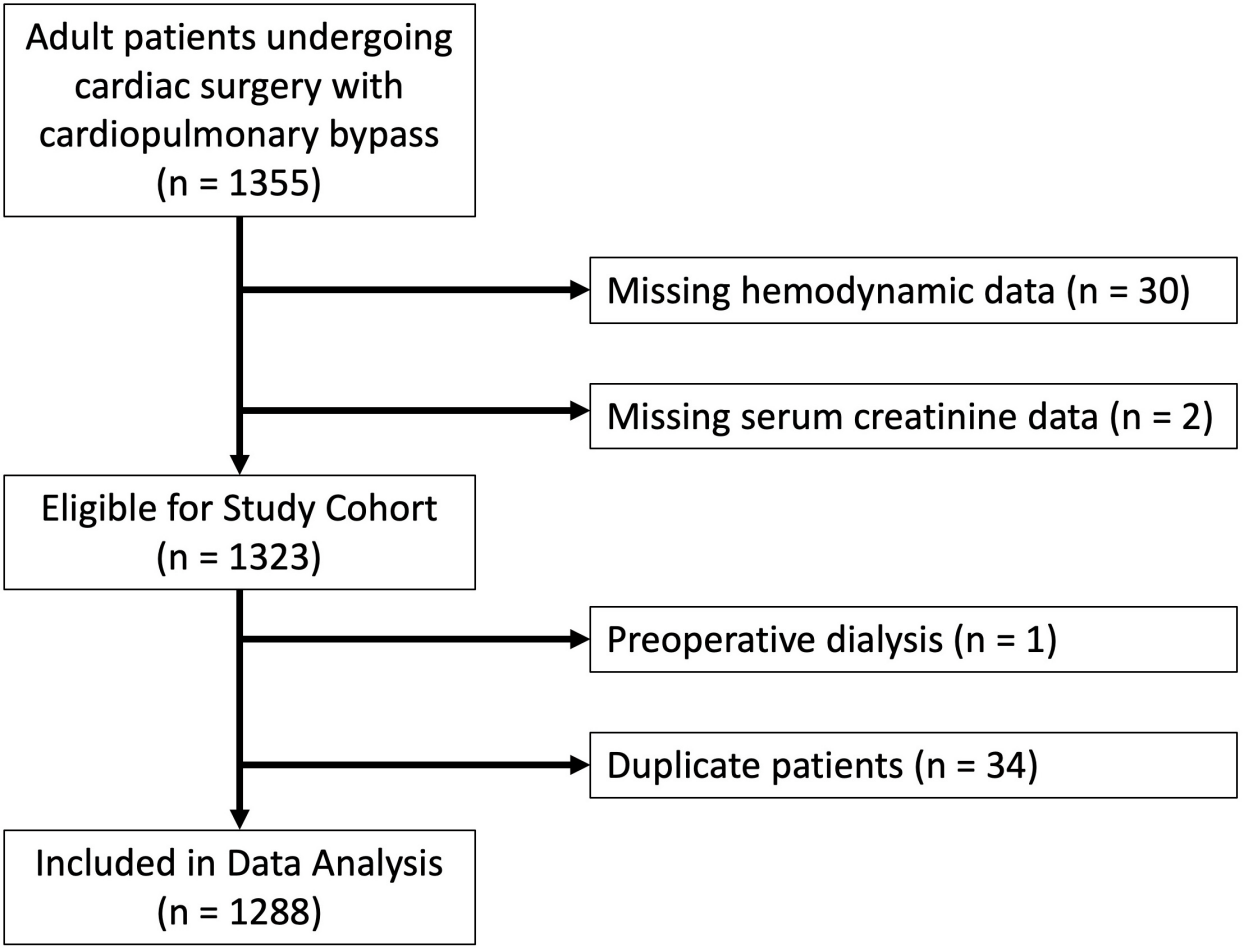
Flowchart of study design with reasons for exclusion for patients excluded from final analysis.

Adult patients undergoing cardiac surgical procedures with the use of cardiopulmonary bypass were included in the study. All patients received general endotracheal anesthesia and a pulmonary artery catheter. The intraoperative anesthetic management was not protocolized, but followed institutional standard practices consisting of midazolam and fentanyl for pre-induction sedation followed by isoflurane, fentanyl, midazolam and vecuronium administration during general anesthesia. Protocolized mechanical ventilation and extubation protocols were followed in the intensive care unit (ICU) (22). This protocol initially utilizes volume control ventilation and PEEP of 5 cm H2O and patients are weaned incrementally to pressure support of 5 cm H2O and PEEP of 5 cm H2O until eventual extubation as able based on blood gas analysis and clinical examination (22). Our ventilator weaning protocol is provided in a Supplementary Figure. For duplicate patients, the earliest procedure was used as the index case, with subsequent cases excluded from analysis. A total of 1,355 patients were initially included in the study. Sixty-seven patients were excluded from our study: 30 patients for missing PAPi or other relevant hemodynamic data; 2 patients for missing preoperative serum creatinine data; 1 patient who was requiring hemodialysis preoperatively; and 34 patients who were duplicate (Figure 1).

All hemodynamic data were acquired from the electronic medical record (Epic Systems, Verona, WI). Invasive central and pulmonary artery pressures were measured with a pulmonary artery catheter and associated patient monitor (IntelluVue, Philips Healthcare, Andover, Massachusetts). Patients obtained at least one daily chest radiograph to monitor correct position of the pulmonary artery catheter and clinical or waveform changes concerning for catheter displacement was addressed by an intensivist immediately available for 24-h per day. Hemodynamic measurement samples for CVP and pulmonary artery pressures were taken at a resolution of four samples per second directly from the monitor, which also accounts for inspiration and expiration cycles with updated waves at a speed of 3 cm/min. PAPi was calculated as [(Pulmonary Artery Systolic Pressure–Pulmonary Artery Diastolic Pressure) / CVP] (15) using the average of the first 48-h of postoperative care for each data point. If patients had their pulmonary artery catheter removed prior to 48-h, the average of the time data available was used. In total, we had 396 unique missing datapoints, which translated to about 0.6% of total datapoints obtained for our study cohort.

The primary aim was association of postoperative PAPi and CVP with postoperative acute renal injury, defined as a rise in postoperative serum creatinine of ≥ 1.5 times the baseline per Kidney Disease Improving Global Outcomes (KDIGO) criteria (23). Secondary aims included association of perioperative PAPi and CVP with need for renal replacement therapy (RRT), hospital mortality, 30-day mortality, total postoperative ventilator hours, total postoperative intensive care unit hours, and hospital length of stay (defined in days from date of surgery to discharge).

## Statistical analysis

Categorical variables were described by frequency and percentage while normally distributed continuous variables were described by mean, minimum, and maximum. For comparisons between groups, Chi-square tests were used to test categorical variables and the Student's *t*-test used to test continuous variables. Correlations were tested using the Pearson correlation. Bivariate logistic regression models were used to calculate odds ratios of development of renal injury and requirement of renal replacement therapy. Two separate multiple logistic regression models were used for each, with one containing PAPi as an independent variable and the other containing CVP as an independent variable due to PAPi being a derived variable from CVP. To better compare odds ratios for our primary aims, we used standardized values of one standard deviation for PAPi and CVP in the regression analysis. All analyses were conducted using SAS V 9.4 (Copyright © 2002-2012 by SAS Institute Inc., Cary, NC, USA. All Rights Reserved). A *p*-value threshold of < 0.05 was used for statistical significance.

## Result

A final cohort of 1,288 patients were included for analysis. Patient characteristics are provided in Table 1. The median time for pulmonary artery catheterization and data collection was 20.3 h. The average postoperative CVP was 10.3 mmHg (Min-Max 1–36.8mmHg) and the average postoperative PAPi was 2.01 (Min-Max 0.29–10.33).

**Table 1:** Patient characteristics and their association with postoperative PAPi and CVP values.

	**Total** **(*N* = 1,288)**	**Postoperative PAPi**		**Postoperative CVP**	
	**Mean** **(min-max) or *N* (%)**	**CC or mean (95% CI)^b^**	***P*-Value**	**CC or mean (95% CI)^b^**	***P*-Value**
Age (years)	62.5 (17–88)	0.19	<0.01	−0.10	<0.01
Sex					
Male (%)	936 (73)	2.11 (2.04–2.19)	<0.01	9.8 (9.6–10.0)	<0.01
Female (%)	352 (27)	1.73 (1.6–1.82)		11.3 (10.9–11.7)	
Race					
Caucasian (%)	1,064 (83)	2.05 (1.98–2.11)	0.02	10.1 (9.9–10.3)	0.02
Black (%)	122 (9)	1.91 (1.73–2.08)		11.1 (10.4–11.8)	
Other (%)	93 (7)	1.79 (1.58–1.93)		10.9 (10.3–11.6)	
Unknown (%)	9 (1)	1.39 (1.18–1.53)		11.4 (10.7–12.1)	
Tobacco use					
Yes (%)	427 (33)	1.98 (1.90–2.05)	0.20	10.3 (10.1–10.5)	0.20
No (%)	861 (67)	2.06 (1.96–2.15)		10.2 (9.9–10.5)	
Chronic lung disease					
Yes (%)	266 (21)	2.14 (1.99–2.29)	0.06	10.7 (10.3–11.1)	0.06
No (%)	1,021 (79)	1.98 (1.92–2.04)		10.1 (9.9–10.3)	
Diabetes					
Yes (%)	481 (37)	2.05 (1.96–2.15)	0.26	10.4 (10.1–10.7)	0.26
No (%)	807 (63)	1.98 (1.91–2.06)		10.1 (9.9–10.4)	
BMI (kg/m^2^)	29.9 (15.5–63.1)	−0.12	0.01	0.24	0.01
Ejection fraction (%)	51 (10–85)	−0.05	0.09	0.01	0.09
Serum creatinine (g/dL)	1.06 (0.33–6.87)	0.04	0.12	0.06	0.12
Postoperative CVP (mmHg)	10.3 (1–36.8)	−0.63	<0.01	n/a	<0.01
Postoperative PAPi	2.01 (0.29–10.33)	n/a		n/a	
Surgery status					
Elective (%)	777 (60)	2.08 (1.99–2.16)	<0.01	10.0 (9.8–10.2)	<0.01
Urgent (%)	414 (32)	1.97 (1.88–2.07)		10.4 (10.1–10.8)	
Emergent (%)	97 (8)	1.63 (1.48–1.79)		11.4 (10.7–12.0)	
Procedure category					
CABG (%)	591 (46)	2.03 (1.95–2.12)	0.02	9.9 (9.7–10.1)	0.02
Valve (%)	195 (15)	2.12 (1.98–2.25)		10.1 (9.7–10.6)	
Aortic (%)	188 (15)	2.00 (1.83–2.17)		10.1 (9.7–10.6)	
CABG + valve (%)	103 (8)	1.96 (1.89–2.21)		10.8 (9.9–11.4)	
OHT (%)	36 (3)	1.57 (1.31–1.84)		12.1 (10.9–13.2)	
LVAD insertion (%)	68 (5)	1.96 (1.72–2.19)		11.0 (10.3–11.7)	
Other (%)^a^	107 (8)	1.84 (1.68–2.00)		11.0 (10.4–11.7)	
CPB Time (min)	109 (7–360)	−0.04	0.14	0.12	0.14
Perioperative IABP use					
Yes (%)	68 (5)	1.74 (1.53–1.95)	0.01	11.9 (11.0–12.8)	0.01
No (%)	1,220 (95)	2.03 (1.97–2.08)		10.2 (10.0–10.3)	
Reoperation					
Yes (%)	283 (22)	2.04 (1.92–2.17)	0.56	10.8 (10.4–11.2)	0.56
No (%)	1,005 (78)	2.00 (1.94–2.07)		10.1 (9.9–10.3)	

a Other procedures: pulmonary thrombectomy, cardiac tumor removal, subaortic membrane removal, epicardial lead placement.

b Correlation coefficient used for continuous variables and Mean (95% CI) used for discrete variables. For example, male patients had an average postoperative PAPi of 2.11 with a 95% C.I. of 2.04–2.19. The correlation between CPB time (in min) with postoperative PAPi was −0.04, with a p-value of 0.14.

BMI, body mass index; CABG, coronary artery bypass graft; CPB, cardiopulmonary bypass, CVP, central venous pressure; IABP, intra-aortic balloon pump; LVAD, left ventricular assist device; Min, minimum; Max, maximum; OHT, orthotopic heart transplantation; PAPi, pulmonary artery pulsatility index.

The associations between CVP and PAPi with primary and secondary aims are provided in Table 2. Development of postoperative renal injury occurred in 29.8% of patients (*n* = 384). These patients had a lower average postoperative PAPi [1.79 (95% C.I. 1.70–1.87) vs. 2.11 (2.03–2.18), *p* < 0.01] and a higher average postoperative CVP [11.5 mmHg (95% C.I. 11.2–11.9) vs. 9.7 mmHg (95% C.I. 9.5–9.9), *p* < 0.01] compared to patients who did not develop renal dysfunction. Lower postoperative PAPi was also associated with patients who experienced in-hospital mortality [1.59 (95% C.I. 1.28–1.91) vs. 2.02 (1.96–2.08), *p* = 0.02] and 30-day mortality [1.63 (95% C.I. 1.35–1.90) vs. 2.02 (95% C.I. 1.96–2.08), *p* = 0.03]. Higher CVP was also associated with hospital mortality [14.3 mmHg (95% C.I. 11.7–16.9) vs. 10.2 mmHg (95% C.I. 10.0–1.03), *p* < 0.01] and 30-day mortality [13.6 mmHg (95% C.I. 11.4–15.9) vs. 10.2 mmHg (95% C.I. 10.0–10.3), *p* < 0.01]. Postoperative renal replacement therapy was required in 2% of patients (*n* = 22). These patients had lower postoperative PAPi [1.42 (95% C.I. 1.15–1.70) vs. 2.02 (1.96–2.08), *p* < 0.01] and higher postoperative CVP [14.4 mmHg (95% C.I. 11.9–16.8) vs. 10.2 mmHg (95% C.I. 10.0–10.3), *p* < 0.01]. Lower postoperative PAPi values correlated with increased hospital length of stay (correlation coefficient −0.09, *p* < 0.01), postoperative ventilator hours (−0.13, *p* < 0.01) and ICU hours (−0.11, *p* < 0.01). Likewise, patients with higher CVP values tended to have increased length of stay (0.19, *p* < 0.01), ventilator hours (0.22, *p* < 0.01) and ICU hours (0.30, *p* < 0.01).

**Table 2:** Bivariate analysis of primary and secondary aims by CVP and PAPi.

	**Total** **(*n* = 1,288)**	**Postoperative PAPi**		**CVP**			
	**Mean** **(min-max) or *N* (%)**	**Mean (95% CI)^a^**	**CC or OR^b^**	***P*-value**	**Mean (95% CI)**	**CC or OR^b^**	***P*-value**
Renal injury							
Yes (%)	384 (30)	1.79 (1.70–1.87)	1.64	<0.01	11.5 (11.2–11.9)	1.24	<0.01
No (%)	904 (70)	2.11 (2.03–2.18)			9.7 (9.5–9.9)		
Postoperative RRT							
Yes (%)	22 (2)	1.42 (1.15–1.70)	2.94	<0.01	14.4 (11.9–16.8)	1.29	<0.01
No (%)	1,266 (98)	2.02 (1.96–2.08)			10.2 (10.0–10.3)		
Hospital mortality							
Yes (%)	27 (2)	1.59 (1.28–1.91)	1.89	0.02	14.3 (11.7–16.9)	1.29	<0.01
No (%)	1,261 (98)	2.02 (1.96–2.08)			10.2 (10.0–10.3)		
30-Day mortality							
Yes (%)	32 (2)	1.63 (1.35–1.90)	1.72	0.03	13.6 (11.4–15.9)	1.26	<0.01
No (%)	1,256 (98)	2.02 (1.96–2.08)			10.2 (10.0–10.3)		
Length of stay (days)	7.9 (0–97)	-	−0.09	<0.01	-	0.19	<0.01
Ventilator time (hours)	20.0 (0–717)	-	−0.13	<0.01	-	0.22	<0.01
ICU time (hours)	73.9 (5.4–1,152)	-	−0.11	<0.01	-	0.30	<0.01

a Applicable to discrete variables only.

b Correlation coefficient used for continuous variables and odds ratio used for discrete variables. Odds ratios for PAPi reflect associations with decreasing PAPi.

CVP, central venous pressure; CI, confidence interval; ICU, intensive care unit; Min, minimum; Max, maximum; PAPi, pulmonary artery pulsatility index; RRT, renal replacement therapy.

Our multivariable regression analyses used standardized values of one standard deviation of PAPi (1.0) and CVP (3.0 mmHg) (Table 3). We used OR associated with a decrease in PAPi, which is directly correlated with an increase in CVP, in order to better compare the findings of our models. For renal injury, our regression model containing PAPi as an independent variable demonstrated higher odds of developing renal injury with decreasing PAPi (OR1.39, *p* < 0.01), higher age (OR 1.02, *p* < 0.01), presence of chronic lung disease (OR 1.91, *p* < 0.01) higher BMI (OR 1.04, *p* < 0.01), longer CPB time (1.01, *p* < 0.01), IABP use (1.98, *p* < 0.02), urgent (2.04, *p* < 0.01) or emergent (2.37, *p* < 0.01) surgery, and undergoing VAD placement (3.04, *p* < 0.01) or orthotopic heart transplant (3.69, *p* < 0.01). Similar findings were noted in the regression analysis containing CVP as an independent variable, with higher CVP associated with higher odds of renal injury (1.56, *p* < 0.01).

**Table 3:** Multivariable logistic regression analyses for renal injury and renal replacement therapy in association with postoperative pulmonary artery pulsatility index (PAPi) and central venous pressure (CVP) measurements.

	**Renal injury**				**Renal replacement therapy**			
	**PAPi model^a^**		**CVP model^b^**		**PAPi model^a^**		**CVP model^b^**	
	**OR (95% CI)**	***P*-value**	**OR (95% CI)**	***P*-value**	**OR (95% CI)**	***P*-value**	**OR (95% CI)**	***P*-value**
PAPi (decreasing)	1.39 (1.19–1.64)	<0.01	-	-	1.89 (0.81–4.45)	0.14	-	-
CVP (mm Hg)	-	-	1.56 (1.36–1.80)	<0.01	-	-	1.49 (1.07–2.07)	0.02
Age (years)	1.02 (1.00–1.03)	<0.01	1.02 (1.01–1.03)	<0.01	0.97 (0.93–1.01)	0.17	0.97 (0.93–1.01)	0.13
Sex (female)	1.14 (0.84–1.55)	0.40	1.04 (0.76–1.42)	0.82	1.30 (0.45–3.77)	0.63	1.18 (0.4–3.48)	0.76
Chronic lung disease	1.91 (1.39–2.62)	<0.01	1.74 (1.27–2.39)	<0.01	1.73 (0.52–5.77)	0.37	1.47 (0.43–4.96)	0.54
Diabetes	1.29 (0.96–1.72)	0.09	1.27 (0.95–1.70)	0.11	0.86 (0.26–2.93)	0.81	0.83 (0.24–2.91)	0.77
BMI (kg/m^2^)	1.04 (1.02–1.07)	<0.01	1.03 (1.01–1.06)	<0.01	1.09 (1.00–1.19)	0.04	1.09 (0.99–1.19)	0.06
Ejection fraction (%)	0.99 (0.98–1.00)	0.12	0.99 (0.98–1.00)	0.06	0.99 (0.96–1.04)	0.82	0.99 (0.95–1.04)	0.81
Preoperative creatinine (g/dL)	1.25 (0.87–1.78)	0.22	1.16 (0.81–1.65)	0.42	2.61 (1.31–5.21)	<0.01	2.55 (1.23–5.14)	0.01
CPB time (min)	1.01 (1.00–1.01)	<0.01	1.01 (1.00–1.01)	<0.01	1.02 (1.01–1.03)	<0.01	1.02 (1.01–1.03)	<0.01
Perioperative IABP use	1.98 (1.10–3.58)	0.02	1.79 (0.98–3.28)	0.06	8.30 (2.27–30.3)	<0.01	6.96 (1.81–26.7)	<0.01
Status								
Elective	n/a (index value)	-	n/a (index value)	-	n/a (index value)	-	n/a (index value)	-
Emergent	2.37 (1.27–4.43)	<0.01	2.42 (1.28–4.55)	<0.01	3.98 (0.66–24.1)	0.13	4.09 (0.68–24.5)	0.12
Urgent	2.04 (1.50–2.77)	<0.01	2.00 (1.47–2.73)	<0.01	3.07 (0.84–11.26)	0.09	2.38 (0.65–8.75)	0.19
Procedure category								
CABG	n/a (index value)	-	n/a (index value)	-	n/a (index value)	-	n/a (index value)	-
Aortic	1.05 (0.65–1.70)	0.85	1.03 (0.63–1.67)	0.92	0.77 (0.10–5.82)	0.80	0.56 (0.07–4.37)	0.58
Valve	1.27 (0.82–1.98)	0.29	1.19 (0.77–1.87)	0.43	1.22 (0.15–9.64)	0.85	0.83 (0.10–6.84)	0.86
CABG + valve	1.36 (0.80–2.32)	0.25	1.27 (0.74–2.19)	0.38	3.09 (0.56–17.15)	0.20	1.85 (0.31–10.9)	0.50
LVAD insertion	3.04 (1.44–6.39)	<0.01	2.64 (1.25–5.60)	0.01	3.37 (0.41–27.93)	0.26	2.82 (0.33–24.5)	0.35
OHT	3.69 (1.32–10.3)	0.01	3.15 (1.12–8.85)	0.03	0.36 (0.02–7.31)	0.51	0.25 (0.01–5.39)	0.37
Other^c^	1.05 (0.62–1.80)	0.85	0.96 (0.55–1.65)	0.88	0.92 (0.10–8.76)	0.94	0.55 (0.06–5.23)	0.61
Reoperation	1.01 (0.70–1.45)	0.96	0.96 (0.66–1.38)	0.82	1.48 (0.45–4.90)	0.52	1.59 (0.48–5.29)	0.45

a Standardized unit of one standard deviation for PAPi (1.0) used in regression analysis.

b Standardized unit of one standard deviation for CVP (3.0) used in regression analysis.

c Other procedures: pulmonary thrombectomy, cardiac tumor removal, subaortic membrane removal, epicardial lead placement.

BMI, body mass index; CABG, coronary artery bypass graft; CI, confidence interval; CPB, cardiopulmonary bypass, CVP, central venous pressure; IABP, intra-aortic balloon pump; LVAD, left ventricular assist device; Min, minimum; Max, maximum; OHT, orthotopic heart transplantation; OR, odds ratio; PAPi, pulmonary artery pulsatility index.

**Figure 2:**
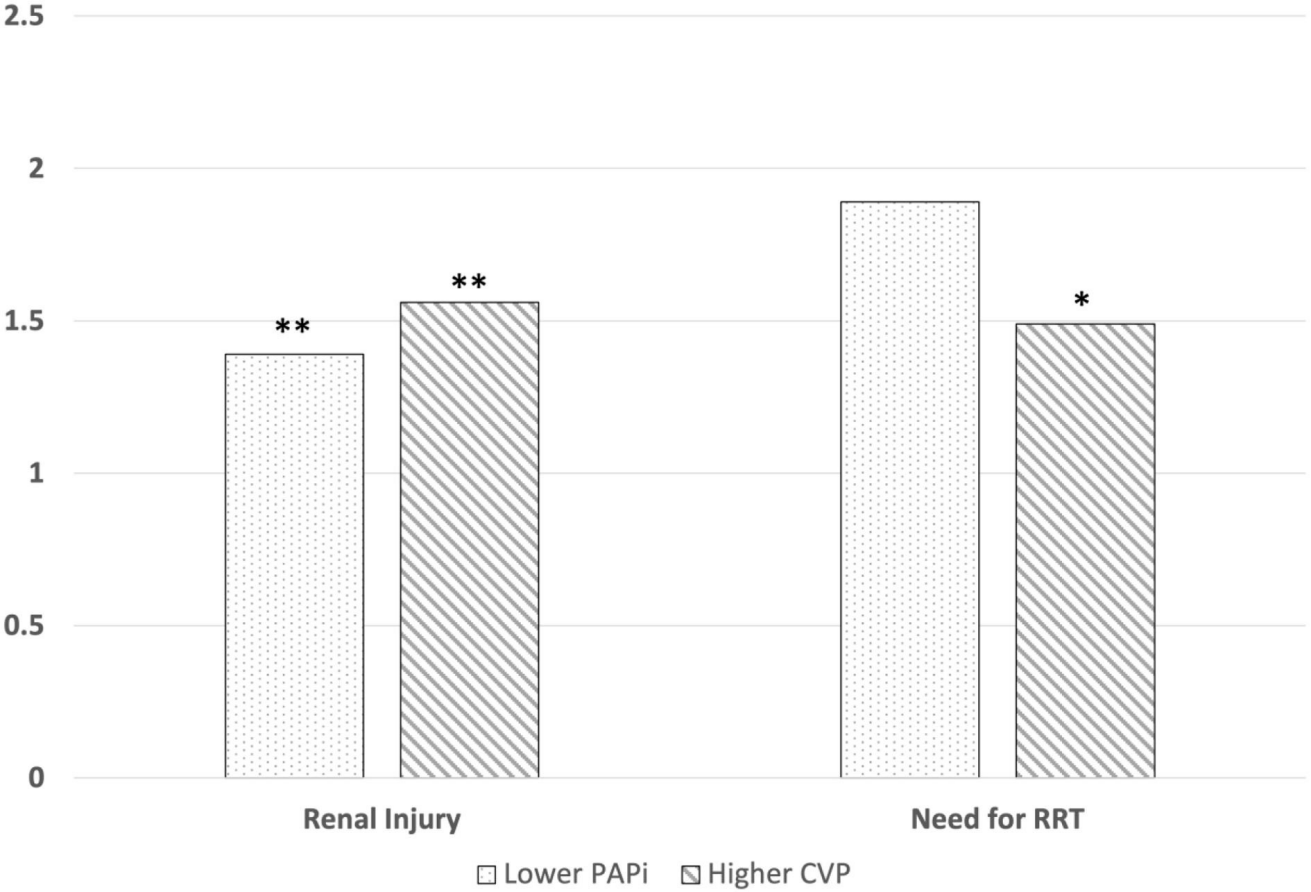
Graphical representations for adjusted odds ratios for the association between postoperative PAPi and CVP and developing CS-AKI and requiring RRT The adjusted odds ratio for developing post-cardiac surgical acute kidney injury (AKI) in patients who developed AKI was 1.39 (*p* < 0.01) for PAPi and 1.56 for CVP (*p* < 0.01). The adjusted odds ratio for requiring RRT in 1.89 (*p* = 0.14) for PAPi and 1.49 for CVP (*p* = 0.02). ** = *p* < 0.01, * = *p* < 0.05.

In our logistic regression for postoperative need for renal replacement therapy (Table 3), our model with CVP as the independent variable demonstrated that higher CVP (OR 1.49, *p* = 0.02) was associated with higher odds ratio of requiring RRT, along with higher preoperative creatinine (2.55, *p* = 0.01), longer CPB time (1.02, *p* < 0.01) and perioperative IABP use (6.96, *p* < 0.01). Our model with PAPi as the independent variable did not demonstrate a significant association with need for RRT (1.89, *p* = 0.14). A graphical representation of adjusted OR for renal injury and need for RRT are visually represented in Figure 2.

## Discussion

This is the first study to demonstrate that both high CVP and low PAPi in the postoperative setting is a risk factor for CS-AKI. The relationship of CVP to AKI has only recently been demonstrated in this population, so corroborating this finding is important in and of itself (10). However, to demonstrate that PAPi, presumably by virtue of reduced right heart function and subsequent venous congestion, is also associated with CS-AKI is a novel finding. Secondly, higher postoperative CVP values were associated with use of RRT, whereas lower PAPi was not. Lastly, we demonstrate that when comparing CVP to PAPi in patients who developed CS-AKI, CVP had a more sensitive odds ratio, potentially making CVP the more important hemodynamic parameter when attempting to prevent CS-AKI.

We chose to standardize values for our analysis given the inherent challenge in directly comparing per-unit differences between PAPi, which is dimensionless, and CVP, which is measured in mmHg. In doing so, CVP proved more clinically valuable than PAPi when comparing adjusted odds ratios of developing CS-AKI. Our findings suggest that PAPi may not have a robust association with CS-AKI compared to CVP, and adds credence to the currently utility of these hemodynamic data points as markers for the risk of developing kidney injury in the postoperative cardiac surgical period (20). This may be due to the process associated with the development of CS-AKI being more dependent upon venous congestion, which is more accurately represented by CVP and less a sequela of PAPi, which represents multiple aspects of right ventricular afterload and may not be as specific. The development of the PAPi calculation was proposed as an easily obtainable and since validated indicator for right ventricular dysfunction (15). Lower PAPi values, commonly < 2.0, have been associated with worse patient outcomes in various cohorts (7, 14, 17–20). While calculation of PAPi relies upon CVP, the two are different in what is represented. PAPi is meant to holistically account for the complex interplay between right heart preload and afterload. Since PAPi is a derived value, changes in PAPi can be driven by its components and may not fully reflect right heart function across a diverse patient population.

Meanwhile, multiple studies have proposed that venous congestion is a contributor to perioperative AKI due to increased renal afterload from the “back up” of volume into the renal glomeruli (24–26). A CVP of >10 mmHg at the conclusion of cardiac surgery has been associated with more than a five-fold increased risk of AKI and more than a four-fold increased risk of mortality (27). Recent studies have also found that both the intraoperative magnitude and duration of increased CVP is associated with increased AKI (10). Thus, using CVP rather than PAPi may be a more appropriate harbinger for CS-AKI rather than PAPi when used in this context and places import upon therapeutics aimed at fluid removal in the ICU (28, 29). These findings may be of interest for advocates for the adoption of less invasive assessments of cardiac function if a central venous catheter can provide adequate clinical information vs. a pulmonary artery catheter (30, 31).

The current study averaged CVP and PAPi values in the ICU, as opposed to hemodynamic values obtained at single timepoints perioperatively. The relationship between CS-AKI and intraoperative and patient factors is well-established in the literature, but the impact of early postoperative care on renal injury is less understood (1–4, 32). However, elevated postoperative CVP is also associated with CS-AKI, and the importance of maintaining adequate hemodynamics for renal perfusion does not diminish in the postoperative setting (6, 27, 32). In addition, recent studies have suggested that a cumulative venous congestion load (i.e., exposure higher CVP for longer lengths of time) is also predictive of CS-AKI (10). We believe our study offers more scrutiny of the postoperative renal load experienced by patients, and the how to better interpret the plethora of hemodynamic variables available to the intensive care team to guide high-quality care.

This study had several limitations. First, this study was a single-center retrospective cohort and as such requires validation in a prospective trial. Additionally, while the majority of intraoperative and postoperative care was protocolized, provider bias regarding individual patient management may still remain. We did not specifically standardize the duration of mechanical ventilation, which is a known confounder to right heart pressures. However, all patients received the same protocolized ventilator management, and neither CVP nor PAPi were strongly correlated with duration of mechanical ventilation (Table 2). We did not specifically examine the role of other confounders such as administration of nephrotoxic agents, inotropic use, hypotension, metabolic acidosis, or diuresis. These potential confounders would certainly affect renal risk and thus, our findings should be considered exploratory until a prospective trial can better elucidate the associations between PAPi, CVP, and renal dysfunction. Due to the large quantity of data obtained (>63,000 individual datapoints for the final analysis cohort), it is hopeful that the impact of such rare confounders is negligible. Additionally, there may have been competitive risk between our outcomes, in particular renal injury and mortality, which did the study did not specifically control for. However, 97% of patients (*n* = 26) who experienced in-house mortality also experienced renal injury based KDIGO criteria, which likely mitigated this risk within our study cohort.

In conclusion, the importance of identifying which patients will suffer from CS-AKI is imperative to prevent and to allow for early management of this comorbid condition that increases postoperative mortality. The current study demonstrates that both lower PAPi and higher CVP values are both associated with the development of CS-AKI. When changes in values are standardized however, a per-unit increase in CVP was more closely associated with development of CS-AKI when compared with lower per-unit decrease in PAPi. This is likely due to CS-AKI being more closely related to venous congestion, regardless of etiology, than to pulmonary factors affecting right ventricular function. Higher CVP was also independently associated with need for postoperative renal replacement therapy. Further investigation is needed to identify if the less invasively obtained value of CVP is a more important predictor for CS-AKI than PAPi.

## Data availability statement

The raw data supporting the conclusions of this article will be made available by the authors, without undue reservation.

## Author contributions

JW and BF: study design and manuscript editing. JW, AH, and VL: data collection. JW and NN: data analysis. JW, AH, NN, and BF: manuscript drafting. All authors contributed to the article and approved the submitted version.

## Conflict of interest

The authors declare that the research was conducted in the absence of any commercial or financial relationships that could be construed as a potential conflict of interest.

## Publisher's note

All claims expressed in this article are solely those of the authors and do not necessarily represent those of their affiliated organizations, or those of the publisher, the editors and the reviewers. Any product that may be evaluated in this article, or claim that may be made by its manufacturer, is not guaranteed or endorsed by the publisher.
