# Supplementary material for: Comparing the associations of central venous pressure and pulmonary artery pulsatility index with postoperative renal injury
Source: Front Cardiovasc Med. 2022 Oct 12;9:967596. doi: 10.3389/fcvm.2022.967596 (PMC9596935; doi:10.3389/fcvm.2022.967596)
Supplement: Supplementary file 1 [file Data_Sheet_1.PDF]

Supplemental Figure 1: Cardiothoracic ICU Ventilation Weaning Protocol

INITIATION AND MAINTENANCE

PATHWAY

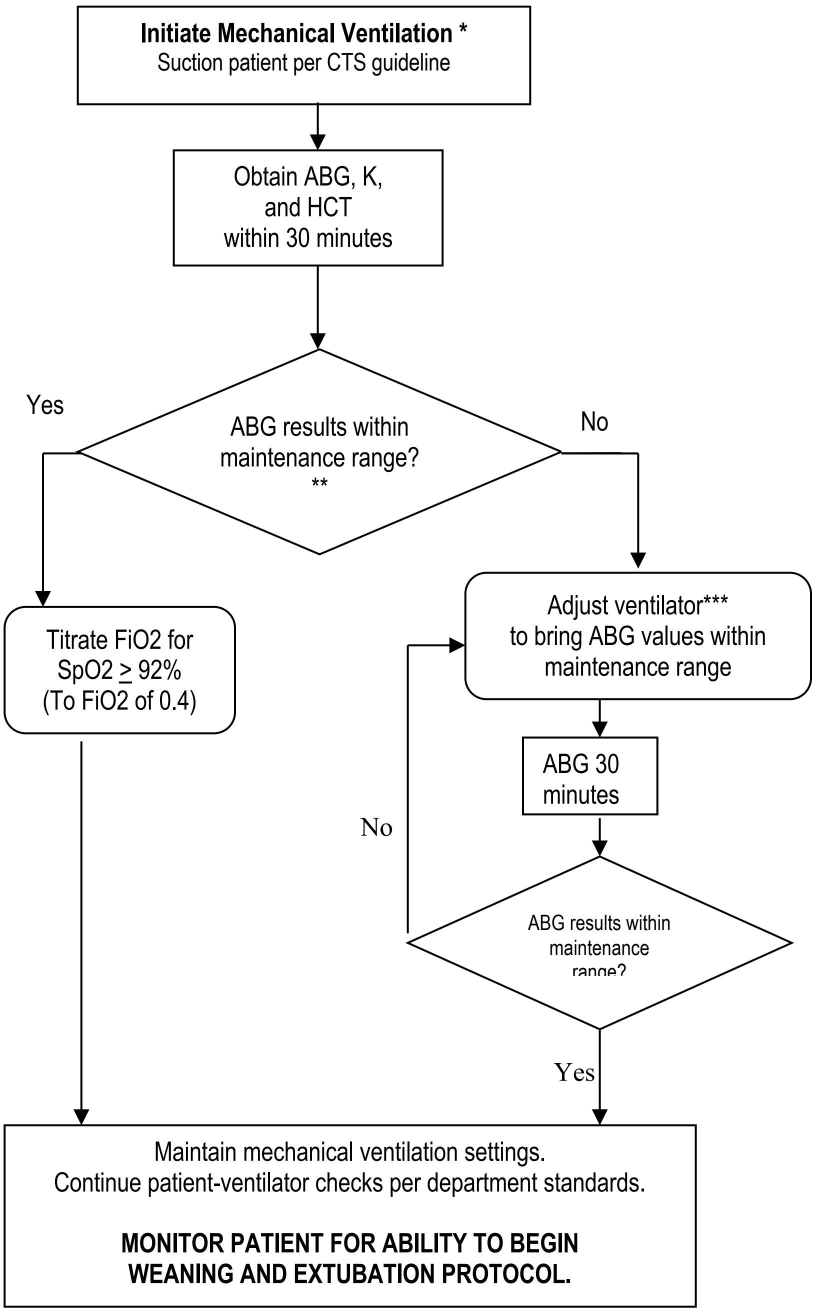

CLINICAL INFORMATION

* INITIAL SETTINGS: (Alarms per department standard)
Assist Control

- R.R. 15 (range 14 – 18)
- Vt 6 -10mL/kg I.B.W.
- PEEP 5 cmH2O (range 5 - 10cmH2O) (notify physician if you increase PEEP over 7)
- FiO2 1.0 (range 0.4 to1.0 – keep SpO2 >92%)
- Pause none

IBW (male) = 50+2.3(ht. inch-60)
(female) = 45.5+2.3(ht. inch-60)
(2.54cm = 1 inch)

** MAINTENANCE ABG RANGE:

pH	7.36- 7.48
PaCO2	35 – 42mm Hg (Target 40)
PaO2	>70mmHg or
SpO2	$\geq$92%

<u>*VENTILATOR REFERENCE:</u>**

<u>Hypoventilation: pH low, PaCO2 high</u>
Respiratory rate may be changed; range 14 - 18.
Tidal Volume may be changed:

a. Vt should remain inside 6-10 mL/kg IBW.
b. The increased tidal volume should not result in plateau pressure above 30 cmH2O

<u>Hyperventilation: pH high, PaCO2 low</u>
Assess for extubation protocol.
Respiratory rate may be decreased; range 8-14
Tidal volume may be decreased. (Vt= 6-10mL/kg IBW)

$$Ve = \frac{\text{present Ve X present PaCO2}}{\text{desired PaCO2}}$$

Notify physician if the third set of ABG's fall outside the maintenance range. Notify physician about any and all concerns.

WEANING AND EXTUBATION

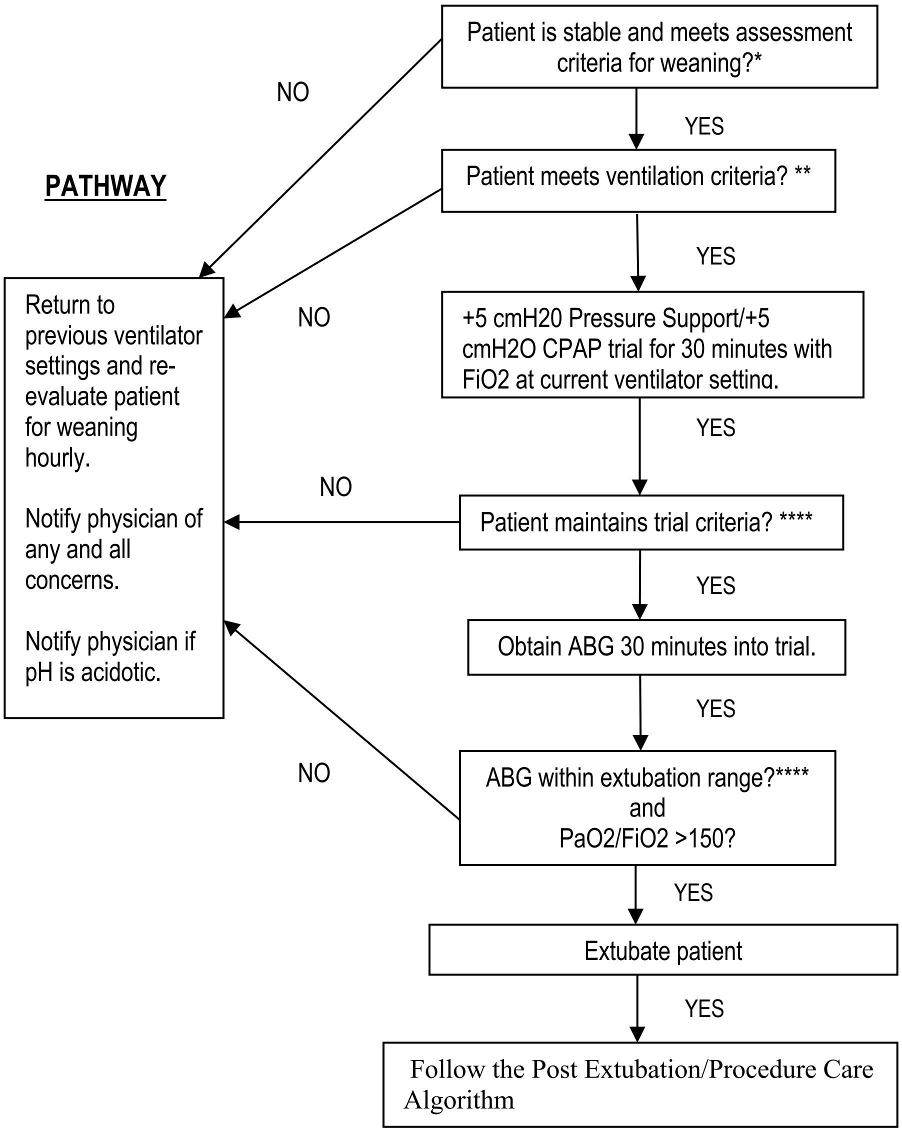

CLINICAL INFORMATION

***ASSESSMENT CRITERIA**
Alert and follows commands
Able to lift head off of bed
Small or moderate amount of sputum
Stable pulse rate
Stable cardiac rhythm
Stable blood pressure
Balloon pump-contact MD for direction
< 200cc/hr combined chest tube drainage over the last 2 hours
Airway leak present when cuff down
PEEP 5cm H2O
PaO2/FiO2 ratio >150

****VENTILATION CRITERIA**
RR > 6 and < 25
MIP > 20 cm H2O
RSBI < 100 (RR/Vt=RSBI)

******TRIAL CRITERIA**
RSBI < 100
RR < 35
SpO2 > 92%
HR < 140 beats/minute
HR <20% increase or decrease from baseline
No dysrhythmia present
Systolic BP < 180 mmHg and > 90mmHg
No anxiety or diaphoresis from increased WOB

******ABG EXTUBATION RANGE**
pH 7.33-7.48
PaCO2 33-48mmHg
PaO2 >70 mmHg
